# Research Progress on Anthocyanin-Mediated Regulation of ‘Black’ Phenotypes of Plant Organs

**DOI:** 10.3390/cimb45090458

**Published:** 2023-09-01

**Authors:** Fei Wang, Jinliao Chen, Ruonan Tang, Ruixin Wang, Sagheer Ahmad, Zhongjian Liu, Donghui Peng

**Affiliations:** Key Laboratory of National Forestry and Grassland Administration for Orchid Conservation and Utilization, College of Landscape Architecture and Art, Fujian Agriculture and Forestry University, Fuzhou 350002, China; wangfei_icon@163.com (F.W.); fjchenjl@126.com (J.C.); trn7020@163.com (R.T.); 13559107013@163.com (R.W.); sagheerhortii@gmail.com (S.A.)

**Keywords:** color patterns, plant organs, anthocyanins, structural genes, transcription factors

## Abstract

The color pattern is one of the most important characteristics of plants. Black stands out among the vibrant colors due to its rare and distinctive nature. While some plant organs appear black, they are, in fact, dark purple. Anthocyanins are the key compounds responsible for the diverse hues in plant organs. Cyanidin plays an important role in the deposition of black pigments in various plant organs, such as flower, leaf, and fruit. A number of structural genes and transcription factors are involved in the metabolism of anthocyanins in black organs. It has been shown that the high expression of R2R3-MYB transcription factors, such as *PeMYB7*, *PeMYB11*, and *CsMYB90*, regulates black pigmentation in plants. This review provides a comprehensive overview of the anthocyanin pathways that are involved in the regulation of black pigments in plant organs, including flower, leaf, and fruit. It is a great starting point for further investigation into the molecular regulation mechanism of plant color and the development of novel cultivars with black plant organs.

## 1. Introduction

The significance of color diversity is recognized in the coevolution between plants and pollinators, such as insects and birds [1,2]. The variety of colors of the flowers of *Delphinium*, ranging from white to pink, scarlet, blue and purple, gives this plant great ornamental potential [3]. In *Chrysanthemum indicum*, a yellow flower is a good source of usual quercitrin and myricetin, which is important for the development of possible pharmaceuticals [4]. Except for the vibrant and vivid color patterns produced by most of the plants, a dark color can also be seen in some plant organs. For example, ‘Queen of Night’ (horticultural hybrid tulip) and *Lisianthius nigrescens* produce flowers with a dark purple color [5]. *Prunus cistena* ‘Pissardii’ possess black leaves [6] and *Aronia melanocarpa* produce black berries [7]. Color is an essential trait of plants, and the ornamental plant cultivars with multiple colors will be more diversified in the future. In particular, novel plant varieties with unique colors will increase in popularity. Therefore, understanding the mechanism of plant color patterns will be useful for breeding plants with a wide range of colors and for studying plant evolution [8,9,10,11].

Studies have found that various colors of plant organs are generally caused by the types and amount of accumulation of specific flavonoids, carotenoids, and alkaloids [12,13,14]. Anthocyanins are among the most important flavonoid compounds that are commonly found in numerous plants and fruits and play a vital role in the pigmentation of plant organs [12,15]. For instance, anthocyanins significantly affect the color of the fuchsia flower of chrysanthemum, the dark purple fruit of eggplant and the pink flower of lily [16,17,18]. The color of reddish leaf in poinsettia, and red and black berries in grape exhibit a significant correlation with the accumulation of anthocyanins [19,20].

Previous studies have shown that the variation in gene expression in the flavonoid biosynthesis pathway leads to a distinct accumulation of anthocyanin in plant organs, resulting in color polymorphism [21]. The reduction of cyanidin accumulation during fruit maturation in *Ananas comosusis* due to downregulation of *AcHOX21* and *AcMYB12*, and the fluctuations in the endogenous levels of JA (Jasmonic acid), GA3 (Gibberellic acid) and auxins drive the discoloration of *A. comosus* peel due to anthocyanin-mediated discoloration [22]. The *NsMYB1* gene promotes the accumulation of anthocyanin in the black fruit of *Nitraria sibirica* Pall. [23]. The purple leaves of *Dendrobium bigibbum* are associated with *MYB2*, and the transient overexpression of *DbMYB2* significantly enhances anthocyanin accumulation in tobacco [24]. In evergreen azaleas, a diverse range of anthocyanins can be observed in purple flowers in contrast to red flowers, while no anthocyanins are detected in the white petals [25]. Moreover, the dark color is attributed to the accumulation of anthocyanin in both the embryos and the seed coats of *Glycine max* [26]. These research findings suggest that the intensification of color is strongly associated with an increase in levels of anthocyanin. Moreover, gibberellins, sugars and light are crucial elements that are necessary for the activation of anthocyanin gene transcription and the accumulation of pigments [27]. The presence of sunlight can enhance the absorption of anthocyanins, particularly in the skin of apples and grapes, while the absence of light can cause the opposite effect [28,29].

The presence of black color in plants is a rare and attractive characteristic, and there exist some studies that have examined the molecular basis of this color in plants. This review examines the studies on the accumulation of anthocyanins and the regulation metabolism, which are responsible for the dark colors in plants. It also broadens our comprehension of the black color patterns found in various plant parts.

## 2. Synthesis Pathways and Regulation of Anthocyanin Metabolism

### 2.1. Biosynthesis of Anthocyanin

Anthocyanins are in the forms of anthocyanidin glycosides, which endow a variety of colors to plant organs, mainly ranging from red to purple and blue [30,31,32,33]. The colors provide plants with distinct visual effects through diverse biosynthetic pathways (examples in Table 1). Previous studies have shown that anthocyanins are derived from a branch of the flavonoid metabolism pathway in plants, and their biosynthesis takes place in three distinct phases [34,35,36].

Stage 1: Phenylalanine → 4-coumaryl-CoA. The primary enzymes, namely phenylalanine ammonia lyase (*PAL*), cinnamate 4-hydroxylase (*C4H*), and 4-coumaroyl-CoA ligase (*4CL*), catalyze the synthesis of phenylalanine, thereby generating 4-coumaroyl-CoA, which serves as the primary substrate for plant anthocyanin biosynthesis. This process is common to many secondary metabolisms in plants [37].

Stage 2: 4-coumaryl-coA and malonyl-CoA → dihydrokaempferol. In this stage, the synthesis of dihydrokaempferol is catalyzed by three different enzymes, namely *CHS*, *CHI*, and *F3H*. This process is a pivotal reaction in flavonoid metabolism, and the genes responsible for the synthesis of these three enzymes are referred to as early biosynthetic genes (EBGs) [12,38].

Stage 3: dihydrokaempferol, dihydroquercetin and dihydromyricetin → various anthocyanins. The enzyme dihydroflavonol 4-reductase (*DFR*) catalyzes the production of dihydrokaempferol, dihydroquercetin, and dihydromyricetin, thereby generating the corresponding leucoanthocyanidins. Then, the leucoanthocyanidins are transformed into anthocyanins with the catalytic action of anthocyanidin synthase (*ANS*) and UDP-glucose flavonoid glucosyltransferase (*UFGT*). This synthesis stage of anthocyanins is represented by the genes that regulate the synthesis of *DFR*, *ANS* and *UFGT*, which are referred to as late biosynthetic genes (LBGs) [39].

**Table 1:** Components of common anthocyanins and their coloration in different organs of plants.

Plants	Main Anthocyanins	Color	Plant Organ	Reference
*Lisanthius nigrescense*	delphinidin-3-*O*-rhamnol(1–6)galactoside, delphinidin-5-*O*-glucoside	black	corolla	[5]
*Cosmos atrosanguineus*	cyanidin-3-*O*-glucoside, cyanidin-3-*O*-rutinoside	black	flower	[40]
*Cercis canadensis*	cyanidin-3-glucoside and malvidin-3-glucoside	purple	flower	[41]
*Dahlia variabilis*	cyanidin-3-(6″-malonylglucoside)-5-glucoside	black	flower	[42]
*Cyclamen purpurascens*	cyanidin-3-*O*-rutinoside, cyanidin-3-*O*-glucoside, delphinidin-3-*O*-glucoside, malvidin-3-*O*-glucoside, peonidin-3-*O*-rutinoside	red	flower	[43]
*Phacelia campanularia*	phacelianin(dicaffeoyl anthocyanin): 3-*O*-(6-*O*-(4′-*O*-(6-*O*-(4′-*O*-β-d-glucopyranosyl-(*E*)-caffeoyl)-β-d-glucopyranosyl)-(*E*)-caffeoyl)-β-d-glucopyranosyl)-5-*O*-(6-*O*-malonyl-β-d-glucopyranosyl)delphinidin	blue	flower	[44]
*Loropetalum chinense* var. *rubrum*	petunidin-3,5-diglucoside	dark purple	leaf	[45]
eggplant	delphinidin-3-*p*-coumaroyl-rutinoside-5-glucoside	dark purple	fruit	[17]
*Crataegus maximowiczii*	cyanidin-3-*O*-glucoside, cyanidin-3-*O*-galactoside	black	fruit	[46]
soybean	cyanidin-3-glucoside and delphinidin-3-glucoside	black	seed	[47]
*Zea mays* L. *sinensis kulesh*	pelargonidin-3-*O*-glucoside	black	seed	[48]

### 2.2. Regulation of Anthocyanin Metabolism

The distribution of anthocyanins varies based on plant species, plant tissues, developmental stages, and environmental factors [49]. Anthocyanins are water-soluble compounds that are produced in the cytoplasm and subsequently transported to the vacuole and other parts of plants [50,51,52]. Until now, the membrane transporters involved in the anthocyanin transport have been confirmed, including ATP-binding cassette, multidrug and toxic compound extrusion (MATE), bilitranslocase homolog (BTL), and vesicle-mediated transport [53]. Although the major transporters have been adequately identified, further investigation is required to determine the molecular mechanism of anthocyanin transport from the synthesis site to the storage site.

The accumulation of anthocyanin in plants is regulated by a series of structural genes [54]. During the development of *Malus hupehensis*, the color of flowers undergoes a transition from red to white due to a decrease in the expression of anthocyanin biosynthesis genes [55]. In the rose variety ‘Rhapsody in Blue’, transient overexpression of *RhF3′H* and *RhGT74F2* has a significant impact on the accumulation of anthocyanins in the blue-purple petals [56]. The expression of two *CsUFGTs* genes exhibits a positive correlation with the substantial accumulation of anthocyanin compounds in the purple-leaf tea plant [57]. Moreover, it has been demonstrated that the expression profiles of *CHS*, *F3H*, *DFR*, *ANS*, and *UFGT* exhibit a positive correlation with the accumulation of anthocyanin in apples [28,58]. However, the expression profiles of these genes vary in plants based on tissue types, growth stages, and varieties.

Three transcription factor families, including MYB, bHLH, and WD40, play a crucial role in the regulation of anthocyanin accumulation [59]. The majority of MYB transcription factors exert a positive influence on the biosynthesis of anthocyanin in plants [60,61]. However, *CmMYB7* is a negative regulator of anthocyanin biosynthesis in ‘Jinba’, a white flowering chrysanthemum cultivar [62]. The *CPC* (Cross-Pathway Control Protein), which is closely associated with epidermis development, has the ability to decrease anthocyanin content in plants through inhibiting the expression of LBGs in the anthocyanin synthesis pathway [63]. The decrease in anthocyanin content observed in *Petunia hybrida* is attributed to the overexpression of *PhMYB27*, which has the ability to prevent the formation of MBW complexes or convert activation complexes into repressive complexes [64].

Numerous studies have revealed that the production of anthocyanin is significantly affected by pH, sugars, temperature, sunlight, and other factors [65,66,67]. The color of anthocyanins is dependent on the pH of the solution; this is because of the molecular structure of anthocyanins having an ionic nature [68]. Under acidic conditions, some of the anthocyanins appear red. Anthocyanins have a purple hue in neutral pH while the color changes to blue in an increasing pH condition [33]. Decreases in orchard temperatures result in a change in the color of the apple pericarp, indicating that the temperature has an impact on the biosynthesis of anthocyanin [69,70]. For example, the presence of high temperatures has the potential to significantly enhance the expression of numerous genes associated with anthocyanin biosynthesis, including but not limited to *PAL1*, *ANS*, *3GT*, *CHS2*, *UA5*, *DF4R*, *CHI*, *UA3GT2* and *UA3GHT5* in strawberry [71]. Moreover, elevated temperatures can enhance the absorption of anthocyanins from the endoplasmic reticulum to the vacuole by triggering the reactivation of *Mate TT12* genes, further deepening the color of fruit in strawberries. However, high temperatures can also reduce the amount of pigment in fruits by inhibiting the expression of genes and enzymatic activity involved in the production of anthocyanins [72,73,74]. Solfanelli et al. [75] studied the role of sugar in the synthesis of anthocyanins in plants. They found a significantly high expression of *CHS*, *CHI*, *F3H*, *F3′H*, and *FLS* at low concentrations of sucrose, whereas a concentrated sucrose solution only induced the expression of *DFR*, *LDOX*, and *UF3GT*. The photoperiod directly affects the expression of structural genes, which in turn regulate anthocyanin accumulation [76]. Exogenous gibberellin promotes the accumulation of anthocyanins in *P*. *hybrida* corolla by inducing the expression of *CHS* [77].

In addition to this, the molecular modification of anthocyanins can affect the formation of color. The process of glycosylation and methylation of anthocyanins results in a redder hue, whereas the accumulation of acylated anthocyanins results in a highly stable blue hue [78]. The balance between biosynthesis and degradation is what determines the accumulation of anthocyanins in plants [79]. The changes in pH, temperature, co-pigmentation, oxygen, and enzymes may affect the stability of anthocyanins, which is influenced by a variety of factors [80,81]. The occurrence of sporadic accumulation and disappearance of anthocyanin during plant development or changes in environmental conditions suggest that anthocyanin degradation is regulated in accordance with its requirements in plants [82]. High temperature increases the expression of some anthocyanin-degrading genes, such as laccase-9 and laccase-14, and also stimulates anthocyanin degradation by enhancing the activity of POD enzymes [83]. Despite the extensive research conducted on anthocyanin biosynthesis, the knowledge regarding its degradation remains limited [84,85]. The color of fruits, flowers, and leaves in plants holds significant ornamental value as ornamental plant, and economic value as in a variety of agricultural products. So, a comprehensive assessment of anthocyanin degradation may provide new insights into ways to inhibit the process and consequently enhance pigmentation in conditions of low synthesis.

### 2.3. Color Modification

Color is one of the most important characteristics of many plant types. But some plants have limited color ranges because of the genetics of the species, and genetic modification technology is the sole efficacious approach to overcome this limitation [86,87]. For example, through genetic modification, the flower color of *Phalaenopsis* spp. and *Cyclamen persicum* can be changed from pink to light pink, from purple to red or pink, respectively [88,89]. There are violet carnations, roses, and chrysanthemums that have been developed by expressing a petunia, pansy, or campanula flavonoid 3-,5-hydroxylase gene, and genetically modified carnation and rose varieties have been commercialized [90]. In addition, transcription factors regulating the anthocyanin pathway have been identified, and as further knowledge is gained regarding the spatial regulation of flavonoid biosynthesis, there will be potential for the genetic modification of pigmentation patterns in more plants [91,92,93].

## 3. Black Organs in Plants

### 3.1. Black Flower

**Figure 1:**
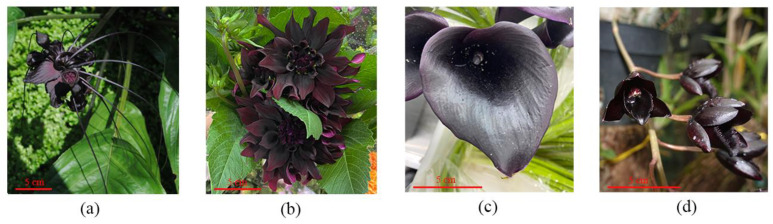
Examples of dark purple flowers. (**a**) Flower of *Tacca chantrieri*. (**b**) Flower of *Dahlia pinnata*. (**c**) Spathe of *Zantedeschia aethiopica*. (**d**) Flower of *Clowesia jumbo*.

The flowers of most angiosperms are bright-colored, which makes them more attractive to pollinators. Despite this, the species that produce black flowers hold a great significance (as illustrated in Figure 1). In fact, there is no plant in nature that is purely black. Although certain plant organs may appear black to the naked eye, they actually possess a dark shade of purple owing to the substantial accumulation of anthocyanins [40,41,46]. In 1996, a variety of *Phalaenopsis aphrodite* with black spots on petals was discovered, which is an important breeding resource for generating color variation in flowering plants [94,95]. *Tulipa Julia* has black patches on the underside of its petals, and the intense violet flowers of ‘Queen of Night’ (a hybrid tulip) appear black under certain lighting conditions [5]. The *L*. *species*, belonging to the Gentianaceae family, is a distinctive black-flowered species in the plant kingdom, renowned for its striking black tubular blossoms that can reach up to 5 cm in length [5]. According to a study conducted by Shibata et al. [96], only five varieties of *Tulipa gesneriana* were found to possess black flowers out of a total of 107 varieties.

### 3.2. Black Leaf and Fruit

**Figure 2:**
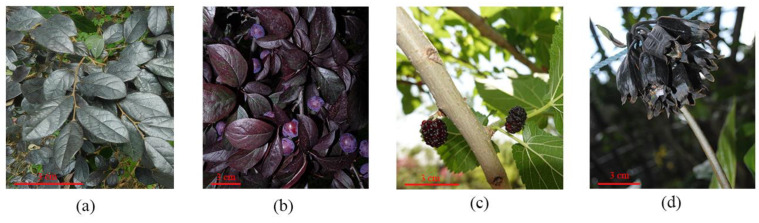
Examples of dark purple leaf and fruit. (**a**) Leaf of *L*. *chinense* var. *rubrum*. (**b**) Leaf and fruit of *P*. *cerasifera*. (**c**) Fruit of *M*. *alba* var. *alba.* (**d**) Fruit of *T*. *chantrieri*.

The majority of plants lack black foliage, but a few species still possess this characteristic, such as *L*. *chinense* var. rubrum and *Prunus cerasifera*, which have dark purple leaves (Figure 2a,b). Black fruits such as *P*. *cerasifera* and *Morus alba* var. *alba*, which possess a high concentration of anthocyanins and appear dark purple (Figure 2b–d), have the potential to serve as effective antioxidants and health supplements [97]. They possess remarkable antioxidant properties in removing free radicals from the body, enhancing blood vessel flexibility, preventing cardiovascular diseases and cancer [98]. The leaves of *P*. *cistena* ‘Pissardii’ and *P*. *cerasifera* exhibt deep purple and aubergine hues, respectively, and possess significant ornamental value [6,99].

In recent years, black fruits have experienced a significant increase in demand owing to their potential utilization as a food colorant and as a source of valuable natural anthocyanins [100]. Black berries (*A*. *melanocarpa*) possess a high level of anthocyanin content, which significantly enhances their nutritional value [7,100,101]. The fruits of *Lycium ruthenicum* and *Morus nigra* are also purple-black or purple-red because they contain abundant anthocyanins [102,103]. Furthermore, there exist vegetables and crops in nature that exhibit a black hue, such as eggplant [17], black carrot [104], black seed soybean [47,105], black rapeseed [37] and black rice [106].

## 4. Regulation of Anthocyanin Metabolism in Black Organs in Plants

### 4.1. Components of Anthocyanins

**Figure 3:**
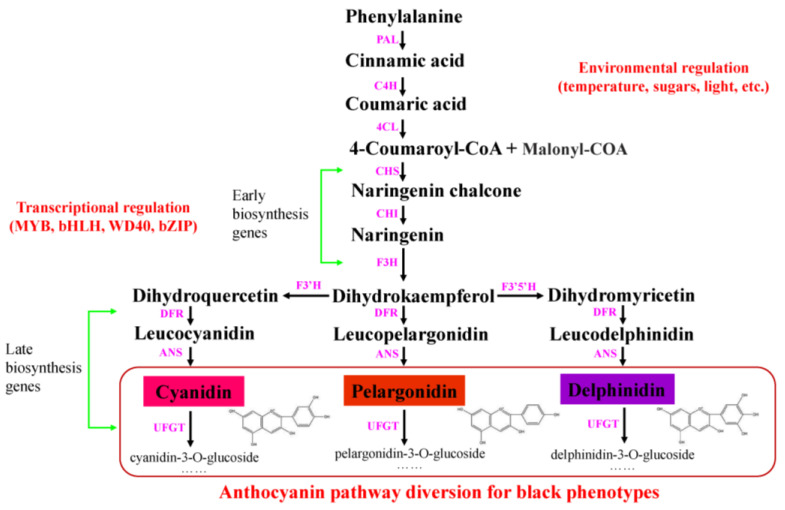
A simplified schematic of the anthocyanin pathway leading to black phenotypes in plant. PAL: Phenylalanine ammonia lyase. C4H: cinnamate 4-hydroxylase. 4CL: 4-coumaroyl-CoA ligase; CHS: Chalcone synthase; CHI: Chalcone isomerase; F3H: Flavanone-3-hydroxylase; F3′H: Flavanone-3′-hydroxylase; F3′5′H: Flavanone-3′,5′-hydroxylase; DFR: Dihydroflavonol-4-reductase; ANS: Anthocyanidin synthase; UFGT: UDP-glucose flavonoid 3-O-glucosyltransferase.

As depicted in Figure 3, some studies have demonstrated that cyanidin, pelargonidin, and delphinidin are the common anthocyanins found in the dark plant organs [107,108,109]. The black flower color of *D*. *variabilis* is caused by the substantial accumulation of cyanidin-3-(6″-malonylglucoside)-5-glucoside [108]. The purple-violet flowers of transgenic chrysanthemum are caused by the accumulation of delphinidin in ray florets, which is caused by the B-ring hydroxylation of anthocyanin, which transforms cyanidin to delphinidin, resulting in the flower color changing from magenta to purple or pink to violet [110]. In the black flowers of *Tulipa* ‘Queen of Night’, three primary anthocyanins are identified, namely delphinidin (50%), cyanidin (29%) and pelargonidin (21%), and delphinidin 3-glucoside is the most common type of delphinidin pigment [96]. The *p*-coumaroyltriglycoside of delphinidin is a predominant constituent of the dark purple flowers of *Viola tricolor* ‘Jet Black’ [111]. The flowers of black *C*. *atrosanguineus* contain two primary anthocyanins, namely cyanidin-3-*O*-glucoside and cyanidin-3-*O*-rutinoside, and the total anthocyanin content in the black variety is approximately 3~4 times higher than that in the red variety [40]. The black corolla of *L*. *nigrescense* contains one major pigment and one minor pigment, and the contents of delphinidin-3-*O*-rhamnol(1–6)galactoside and delphinidin 5-*O*-glucoside account for 24% of the petals’ dry weight [5].

The leaves of *P*. *cistena* ‘pissardii’ appear deep purple when they are exposed to strong sunlight [6]. Coexistence and interaction among cyanidin galactoside, cyanidin and chlorophyll are the main causes of the purplish red leaf of *P*. *cerasifera* [99]. Petunidin-3-*O*-glucoside, anthocyanin-3-*O*-galactoside, and anthocyanin-3-*O*-glucoside are the main anthocyanins that cause the purple leaf phenotype of the tea plant [112]. Zhao et al. [113] have demonstrated that a millet variety (B100) exhibits purple leaves during the seedling and maturity stages. Purple pigments are mainly distributed in the leaf epidermis. The purple leaf color of *L*. *chinense* var. *rubrum* is influenced by the petunidin-3,5-diglucoside [45].

Five cyanidin derivatives have been identified from blackberry, namely cyanidin-3-rutinoside, cyanidin-3-(malonyl)-glucoside, cyanidin-3-xyloside, cyanidin-3-glucoside and cyanidin-3-dioxaloylglucoside [114,115]. A previous study revealed the presence of four significant anthocyanins in the purplish black berries of *A*. *melanocarpa*, including cyanidin-3-*O*-galactoside (68.68%), cyanidin-3-*O*-arabinoside (25.62%), cyanidin-3-*O*-glucoside (5.28%) and cyanidin-3-*O*-xyloside (0.42%) [7]. The purple black fruits of *L*. *ruthenicum* are loaded with petunidin derivatives, which have high ornamental and economic significance [102]. The maturation stage of the mulberry fruit drives the gradual change in color from light red to blackish purple due to the accumulation of anthocyanins. A study conducted on 11 genotypes of *Morus alba*, comprising five black, four white, and two pink multiple fruit varieties, revealed that the abundance of anthocyanins in black fruits ranges from 45.42 to 208.74 mg per 100 g [116], while the amount of cyanidin-3-glucoside in the fresh fruit of *M*. *nigra* was very high, at 704.1 mg per 100 g [103]. The black-colored fruits of *C*. *maximowiczii* are closely associated with the accumulation of cyanidin, pelargonidin, peonidin and delphinidin derivatives, particularly cyanidin-3-*O*-glucoside and cyanidin-3-*O*-galactoside. And the contents of delphinidin-3-*O*-galactoside, pelargonidin-3-*O*-arabinoside, pelargonidin-3-*O*-glucoside, peonidin-3-*O*-arabinoside and peonidin-3-*O*-glucoside in black peel are twice as high as in red peel [46].

Previous studies have revealed that the dark purple of eggplant is attributed to delphinidin-3-p-coumaroylrutinoside-5-glucoside [17], whereas the accumulation of black pigments in soybean seeds is caused by cyanidin-3-glucoside and delphinidin-3-glucoside [47,105]. In *Capsicum annuum*, only a single anthocyanin (delphinidin-3-*p*-coumaroyl-rutinoside-5-glucoside) is found in the violet fruit, black fruit, and black leaves. The distinctive black pigmentation is caused by the high concentrations of delphinidin, combined with chlorophyll and other carotenoid pigments [117]. The dark purple color of *Daucus carota* subsp. *sativus* (a black variety of carrot) is due to an acylated form of anthocyanin, namely [cyanidin-3-(*p*-coumaroyl)-diglucoside-5-glucoside] [104,118]. The content of black pigment in the black rapeseed seed coat is significantly higher than that of other tissues [37]. Four anthocyanins were identified from black rice, namely cyanidin-3-rutinoside, peonidin-3-glucoside, cyanidin-3,5-diglucoside and cyanidin-3-glucoside [106,119].

In general, the genetic background of the species or variety determines the constituents of anthocyanins in plants [120]. Furthermore, certain studies have suggested that the development of plant color is correlated with the structure of organ tissue, pigment distribution and its types. So, it is possible that it will be regulated through genetic engineering, which has made rapid progress in color breeding of plants for its advantages over traditional breeding technologies [12]. Nonetheless, the mechanism of anthocyanin synthesis and metabolism is highly intricate, encompassing numerous metabolic steps and enzymes. Therefore, there exist numerous structural genes and regulatory genes associated with anthocyanin pigments (37–40). At present, scientists have conducted a comprehensive examination of the synthetic pathway of anthocyanins, which are commonly present in plants, and their associated genes. However, it remains challenging to alter a specific trait of plants to generate distinctive color phenotypes of the species and to breed novel varieties with stable inheritance within a brief timeframe.

### 4.2. Structural Genes

The accumulation of pigments in different plant organs is regulated by many structural genes, which are involved in the synthesis of anthocyanins. For example, the knockdown of *F3H* by RNAi in torenia with blue flower produces white flowers [121]. The accumulation of anthocyanins and flavonols in the white and red flower species are caused by the expression of *DFR* and *FLS* genes, and heterologous *FLS* expression in transgenic tobacco promotes flavonol biosynthesis and blocks anthocyanin accumulation, leading to white flowers [122]. In addition, *FNS* and *IVS* are key genes involved in anthocyanin biosynthesis and regulation in black flower plants. In the black flower variety of *D*. *pinnata*, DvIVS-1 promoter has high activity but the expression of *DvFNS* is significantly decreased. Moreover, artificial silencing of *FLS* or *FNS* results in increased accumulation of anthocyanin in *P*. *hybrida* [123]. This suggests that the silencing of *DvFNS* can lead to the loss of flavonoids and eliminates competition for substrates, so that substrates originally used for flavonoid synthesis can be used for anthocyanin synthesis [42]. Then, the DvIVS-1 promoter helps to synthesize large amounts of anthocyanins in black flowers of *D. pinnata*. A high expression of a number of genes (such as *RsCHI1*, *RsFLS1*, *RsANS2*, and *RsAT2*) contributes to the deep blackish crimson flowers of a variety of *Rhododendron sanguineum* [21]. Some research shows that flower color deepens with the increase in anthocyanin content [124,125]. Hence, high expression levels of structural genes are primarily useful for deep hues by promoting the production of anthocyanin [126]. The study on the reddish-purple color in the petals of *Rhododendron simsii* flowers have shown that co-pigmentation, normally with flavonols contributed by *RsFLS*, may result in high accumulation of anthocyanins that shift color toward deep blackish crimson [127].

The expressions of nine genes (*PAL*, *4CL*, *DFR*, *LDOX-1*, *LDOX-2*, *AT*, *UFGT*, *GT*, *5GT*) related to anthocyanin synthesis are significantly higher in purple leaf of foxtail millet (B100) at maturity stage than the green leaf variety (YG1). The expression of three genes (*DFR*, *LDOX-2* and *AT*) in purple leaf of B100 are significantly higher at seedling stage and maturity stage [113]. A previous study confirmed the key structural genes of anthocyanin biosynthesis in purple leaf of ZK, including two *F3′H* genes, two *ANS* genes with positive correlations and three PPO genes with negative correlations [112]. Zong et al. [128] detected the transcriptional product of *AN2* in the black fruit of *Lycium barbarum*. The genetic diversity analysis of *AN2* gene also shows that yellow, white, purple, and red cultivars of *Lycium chinense* originate from *L*. *barbarum*. The overexpression of *IbMYB1-2* can significantly increase anthocyanin content in the root tuber of transgenic sweet potato [129]. In the anthocyanin biosynthesis pathway, the high expression of *F3′H* and *UFGT* genes promotes a high accumulation of cyanidin derivatives, producing *Crataegus pinnatifida* with black fruit [46].

### 4.3. Transcription Factors

The regulation of anthocyanin accumulation is jointly regulated by transcription activators and transcription repressors, primarily comprising MYB, bHLH, WD40, and bZIP [59]. *PeMYB11* is one of them, and it is a major R2R3-MYB TF that is highly expressed in the black flowers of *Phalaenopsis equestris* [95]. The HORT1 (Harlequin Orchid RetroTransposon 1) can lead to a strong expression of *PeMYB11*. Therefore, the flowers of *P.* Yushan Little Pearl variety, which contains HORT1, have an excellent anthocyanin accumulation capacity [95,130]. The purple spots on the sepals of *Phalaenopsis aphrodite* ‘Panda’ are regulated by *PeMYB7*, *PeMYB11*, miR156, and miR858 [130]. Moreover, it should be noted that miR156 and miR858 are the primary siRNAs of *PeMYB7* and *PeMYB11*, respectively, and both cause a significant increase in the expression of genes associated with the anthocyanin biosynthesis pathway (*PeCHl*, *PeANS*, *PeC4H*, *PeF3H*, *PeF3′H*, *Pe3Hl*, and *Pe4CL2*) in spot tissues [130]. In addition, the abnormal expression of bHLH or MYB results in the appearance of dark purple leaf and flower in transgenic petunia plants [131,132]. Anthocyanin accumulation in purple leaves of ZK is strongly correlated with *CsMYB90*, and *CsMYB90* overexpression in transgenic tobacco plants with dark purple callus is also observed [112].

Research has demonstrated that the bZIP family principally functions as a positive regulator of anthocyanin biosynthesis. However, Tu et al. [133] have discovered that *VvbZIP36* is a negative regulator of anthocyanin biosynthesis in *Vitis vinifera* and plays an important role in balancing the synthesis of stilbenes (α-viniferin), lignans, flavonols, and anthocyanins. The insertion of a precursor DNA transposon into the regulatory region of Purple (Pr), which belongs to the R2R3-MYB TF encoding genes, results in the up-regulation of Pr expression, thereby causing the accumulation of dark color in *Brassica oleracea* [134]. It appears that the purple color of *Ipomoea batatas* is caused by the dominant expression of *IbMYB1* [135]. In conclusion, the enhanced expression of regulatory transcription factors in the anthocyanin biosynthesis pathway may be responsible for the appearance of black flowers, leaves, and fruits in plants. However, the specific molecular mechanism is still to be elucidated.

### 4.4. Other Factors

The stability of anthocyanins can be increased by modification to form stable structures. In most plants, only *O*-glycosylation occurs for anthocyanins. In grapes, the structures of the individual anthocyanins include both 3-*O*-monoglucosides and 3,5-*O*-diglucosides. Diglucosidic anthocyanins are more stable than their monoglucosidic counterparts, whereas monoglucosidic anthocyanins tend to have deeper colors than their diglucosidic counterparts [136]. Furthermore, prolonged exposure to high temperatures and prolonged exposure to sunlight significantly affect the stability of anthocyanins in plants [137]. For example, anthocyanin stability against heat stress is increased by the methoxylation and acylation of cyanidin-3-*O*-glucoside from blackberries [138]. Diacylated anthocyanins provide significantly higher blue color stability to red cabbage at 50 °C compared to non-acylated anthocyanins [139]. In black carrot (*Daucus carota* L.), acylated anthocyanins remain more stable during temperature increases of 20–50 °C than non-acylated anthocyanins from blackberry (*Rubus glaucus* Benth.) [140]. And the level of anthocyanins from black carrots remains relatively stable until 90 °C, probably due to di-acylation of the anthocyanin structure [141,142]. Anthocyanins are protected from hydration by acylation, thereby making them more stable, because acylated anthocyanins are generated after the acylation of glycosyl groups of anthocyanins [143]. However, the acylated anthocyanins in black carrot are decomposed under extreme heat stress (95 °C), indicating that the stability of acylated anthocyanins is rapidly decreased [144]. Acylated anthocyanins are found in flowers and vegetables, whereas non-acylated anthocyanins are mostly distributed in fruits [145].

Some encoding enzymes used in biosynthesis and genes responsible for regulating black pigmentation have been identified using advanced molecular biology techniques. But the effect of accumulation and stability on various factors (e.g., pH, anthocyanin transport) deserves further investigation.

## 5. Conclusions and Future Directions

In this review, the molecular mechanism of anthocyanin-mediated black pigmentation in plants is analyzed. Cyanidin is the key factor in black pigmentation and induces black color in ornamental and fruit crops, but the current investigations into black pigmentation in plants are inadequate. Further research on the temporal variation of gene expression in diverse species, organs, and tissues, the interactions between transcription factors and genes, and the effects of anthocyanin transport and of environmental factors on black pigmentation are still lacking. Therefore, it is imperative to select materials of wild-type or self-crossing origin, possessing original color and relatively stable homozygous genotypes, in order to conduct further investigations on the molecular regulation mechanism of black color in plants. It also holds a great significance to achieve color modifications to obtain black color in ornamental plants, fruits, and vegetable crops through the utilization of genetic engineering technology in the future.

## Data Availability

The datasets analyzed in this study are available in the manuscript text.
